# Evaluation of the Turkish Population’s Perspective on COVID-19 Vaccine Hesitancy and Routine Childhood Vaccine Applications: National Survey Study

**DOI:** 10.3390/vaccines11040779

**Published:** 2023-03-31

**Authors:** Sami Akbulut, Gulseda Boz, Ali Ozer, Tevfik Tolga Sahin, Cemil Colak

**Affiliations:** 1 Department of Surgery and Liver Transplant Institute, Inonu University Faculty of Medicine, Malatya 44280, Turkey; 2 Department of Public Health, Inonu University Faculty of Medicine, Malatya 44280, Turkey; 3 Department of Biostatistics, Bioinformatics and Medical Informatics, Inonu University Faculty of Medicine, Malatya 44280, Turkey

**Keywords:** COVID-19, public health, vaccine hesitancy, anti-vaccination, childhood vaccinations

## Abstract

Background: It is important to evaluate the attitude of society towards vaccines to understand the rates of acceptance and hesitance towards vaccination, which are essential components of public health and epidemiology. This study aimed to evaluate the perspective of the Turkish population on COVID-19 status, rate of vaccination, and also to evaluate the reasons for refusal to vaccinate, vaccine hesitancy, and related factors. Methods: A total of 4539 participants were included in this population-based descriptive and cross-sectional study. The Nomenclature of Territorial Units for Statistics (NUTS-II) was used to obtain a representative sample and for this purpose Turkey was divided into 26 regions. Participants were randomly selected based on the demographic features and population ratios of the selected regions. The following parameters were evaluated: sociodemographic characteristics and perspectives on COVID-19 vaccines, Vaccine Hesitancy Scale Adapted to Pandemics (VHS-P), and Anti-Vaccine Scale-Long Form (AVS-LF) questions. Results: A total of 4539 participants, 2303 (50.7%) male and 2236 (49.3%) female, aged between 18 and 73 years, were included in this study. It was observed that 58.4% of the participants had hesitations towards COVID-19 vaccination, and 19.6% were hesitant about all childhood vaccinations. Those who did not have the COVID-19 vaccine, who did not think that the COVID-19 vaccine was protective, and who had hesitation to vaccinate against COVID-19 had significantly higher median scores on the VHS-P and AVS-LF scales, respectively (all *p* < 0.01). Those who did not have their children vaccinated in childhood and who were hesitant about childhood vaccinations, had significantly higher median scores on the VHS-P and AVS-LF scales, respectively (all *p* < 0.01). Conclusion: Although the rate of vaccination for COVID-19 was 93.4% in the study, hesitation to vaccinate was 58.4%. The median score of the scales of those who were hesitant about childhood vaccinations was higher than individuals who did not have any hesitation. In general, the source of concerns about vaccines should be clearly seen, and precautions should be taken.

## 1. Introduction

The coronavirus disease 2019 (COVID-19; SARS-CoV-2) emerged as a severe acute viral pneumonia in Hubei province in China. On 30 January 2020, the World Health Organization (WHO) declared COVID-19 as a public health emergency of international concern, which was a declaration that the situation had become a pandemic and necessary precautions had to be taken immediately. On 11 March 2020, WHO declared the COVID-19 outbreak a global pandemic [1,2,3]. Currently, more than 600 million people have had COVID-19 and approximately 6.5 million people died due to COVID-19 and related complications [4]. Various vaccines have been developed to control the spread of COVID-19 and prevent the development of severe COVID-19 [5]. The first mass vaccination program against COVID-19 began in December 2020, and billions of people worldwide have been vaccinated with safe and effective vaccines since then. The objective results of the studies showed that the vaccines prevented severe disease and COVID-19-related deaths [6,7,8].

Vaccination is the most important part of primary public healthcare. Historically, vaccination is a global success story that saves the lives of millions of people every year [9]. In addition, vaccines are critical to preventing and controlling infectious disease-related epidemics and are an indisputable fundamental human right that is imperative for the promotion of public health. It is obligatory that every government should offer vaccination to citizens free of charge which is important for the promotion of the health of individuals in society [9]. WHO reported that vaccination prevents 3.5–5 million deaths from tetanus, diphtheria, measles, and pertussis each year [9]. Childhood vaccination programs are one of the greatest achievements of modern science [9]. Childhood vaccination programs significantly reduce the morbidity and mortality rates of preventable diseases [8,10,11].

The Strategic Advisory Group of Experts on Immunization (SAGE) was founded by the WHO for vaccination and defines vaccine hesitancy as “delayed acceptance or rejection of the vaccine despite the availability of vaccination services” [12,13]. The WHO has declared vaccine hesitancy as one of the ten leading threats to global health [1,2]. Recently, increasing vaccine hesitancy has been considered a threat to the success of vaccination programs and is thought to be one of the reasons for the increased risk of death from preventable diseases and epidemics [10].

Many factors, such as the novelty of the COVID-19 pandemic and the fact that developed vaccines had a shorter period from production to licensing, resulted in an altered perception of the public through media. This has caused great hesitance towards vaccination for COVID-19 [12,14]. The COVID-19 pandemic has shown us that vaccine hesitancy was higher than expected [14]. Stein et al. [14] analyzed vaccine hesitancy and its spread through mass media. There are many studies on vaccination programs and public perspectives on vaccination. 

It is important to evaluate the public perspective on vaccines because physicians and public health specialists should understand the reasons for anxiety and also, the positive attitudes towards vaccination. The causes of hesitancy may differ among different countries and communities. The aims of the study were to determine relationship between the characteristics of COVID-19 in affected individuals and sociodemographic variables, evaluate the rate and causes of hesitancy to vaccinate for COVID-9 in representative sample of the population.

## 2. Materials and Methods

### 2.1. Type and Place of Research

This population-based survey was organized as a descriptive and cross-sectional study that aims to assess the perspective of Turkish society. In this study, a stratified random sampling method was used based on demographic data such as age, marital status, and education level to represent the society best and minimize the risk of bias. The distribution of the participants was calculated based on the Nomenclature of Territorial Units for Statistics-II (NUTS-II) according to the regions detailed below. 

### 2.2. Population and Sample of Research 

The sample size was calculated according to the total population of Turkey which is 83,500,000, and the number of individuals older than 18 years 60 million as of the beginning of 2021 according to the data of the Turkish Statistical Institute. When appropriate data are entered in the blanks related to target population size (60 million), confidence level (99%), and margin of error (2%) variables in the sample size calculator program (https://www.surveymonkey.com/mp/sample-size-calculator, accessed on 21 September 2021), it was calculated that the minimum sample size should be 4160. A total of 4540 participant candidates were interviewed for this study. 

### 2.3. Inclusion and Exclusion Criteria

Individuals older than or 18 years of age, willing to participate in the study, and able to communicate verbally were included in this study. In addition, foreigners and other individuals who are younger than 18 years of age and who did not know sufficient Turkish to understand or answer the survey questions were excluded from the study.

### 2.4. Determination of the Target Population

The NUTS system was used in the sample selection of the study [15]. Twelve Level-I, 26 Level-II, and 81 Level-III NUTS regions have been defined in Turkey according to the population, geographical structure, regional development plans, fundamental statistical indicators, and socioeconomic development ranking criteria of the provinces [15]. NUTS-II was used in this study according to the recommendations of Eurostat, and regional plans were made for Level 2 regions [15]. The provinces connected to the areas of NUTS-II used in this study were Istanbul, Tekirdag, Balikesir, Izmir, Aydin, Manisa, Bursa, Kocaeli, Ankara, Konya, Adana, Antalya, Hatay, Kayseri, Kirikkale, Kastamonu, Zonguldak, Samsun, Trabzon, Agri, Erzurum, Malatya, Van, Gaziantep, Mardin, and Şanliurfa [15].

### 2.5. Ethics Committee Approval and Financial Support

Before commencing this study, ethics committee approval dated at 19.10.2021 and numbered 2021/2618 was obtained from Inonu University Health Sciences Non-Interventional Clinical Research and Publication Ethics Committee. Verbal consent was obtained from all participants, as stated in the ethics committee documents. All stages of the study were carried out according to the guidelines of the Declaration of Helsinki. This study was reported based on the STROBE (strengthening reporting of observational studies in epidemiology) guidelines to reduce the possibility of bias and improve its overall quality [16]. The financial support required for the study was received from Inonu University Scientific Research Projects Coordination Unit (Project No: TSA-2021-2773). 

### 2.6. Parameters and Scales Used in the Study 

This national survey study was organized by the PRP Research and Consultancy Company, experienced in conducting national surveys in Turkey, and a protocol was signed on how to complete the study. The preparation of the questionnaires, the selection and supervision of the interviewers, the reporting of the results, and the testing reliability of the results were carried out per the ISO 9001/ISO 20,252 quality certificate criteria and Esomar rules. The questionnaire prepared for the study was transferred to the CAPI format and was applied by the interviewers. 

#### 2.6.1. Demographic and Social Characteristics Form

After obtaining verbal consent from all the participants, the interviewers asked all the questions in the questionnaire to the participants via the computer-assisted personal interviewing (CAPI) method. The questionnaire form consisted of three parts in terms of content. The first part consisted of a total of seven questions in which the sociodemographic characteristics were questioned. The second part consisted of 11 questions that evaluated the data on COVID-19 disease, perspectives on COVID-19 vaccines, and general immunization programs. In the third part, the “vaccine hesitancy scale adapted to pandemic (VHS-P)” consisting of 10 questions, and the “anti-vaccination scale—long form (AVS-LF)” consisting of 21 questions were included.

#### 2.6.2. Vaccine Hesitancy Scale Adapted to Pandemic (VHS-P)

The Turkish validity and reliability study of the VHS scale developed by Larson et al. [17] was carried out by Capar et al. [18], and the Turkish version was adapted. The answers given to the VHS-P scale, which consists of five Likert-type questions, are listed as strongly disagree (1 point), disagree (2 points), neither agree nor disagree (3 points), agree (4 points), and strongly agree (5 points). The VHS-P scale consists of 10 items and two sub-dimensions. The first sub-dimension is called “lack of confidence”, and all eight items in this sub-dimension are plainly coded. The second sub-dimension is called “risk”, and two items in this sub-dimension are coded as plain. High scores on the scale indicate high vaccine hesitancy. In the Turkish version of the scale, the factor loads of the items were shown to vary between 0.638 and 0.887. The scale’s Cronbach Alpha reliability and internal consistency coefficient were calculated as 0.901.

#### 2.6.3. Anti-Vaccination Scale—Long Form (AVS-LF)

The AVS-LF scale, consisting of 21 questions, was developed by Kilicarslan et al. [19]. The AVS-LF scale is a 5-level Likert-type scale and the answers are listed as exactly disagree (1 point), disagree (2 points), partially agree (3 points), agree (4 points), and exactly agree (5 points). The AVS-LF scale consists of 21 items and four sub-dimensions. The first sub-dimension of the scale is called “benefits and protective value of the vaccine” and all five items in this sub-dimension are reverse coded. The second sub-dimension is called “anti-vaccination” and all six items in this sub-dimension are plainly coded. The third and fourth sub-dimensions are called solutions for non-vaccination/vaccine hesitancy and legitimization of vaccines, respectively. All five items in these two sub-dimensions are plainly coded. The AVS-LF scale has no calculated cut-off value. High scores indicate that the attitude of the individual toward anti-vaccination is high. The Cronbach Alpha value of the scale was calculated as 0.855.

### 2.7. Statistical Analysis

IBM SPSS Statistics (Statistical Package for Social Sciences version 25.0) was used for the data analysis. Qualitative variables were given as numbers and percentages (%). Shapiro–Wilk Test of normality was applied to the variables containing quantitative data, and it was determined that they did not show normal distribution, and the results were given as median and 95% confidence interval (CI). Mann–Whitney U and Kruskal–Wallis analysis of variance was used to evaluating quantitative data that were not normally distributed. After the Kruskal–Wallis H test showed a significant difference, multiple comparisons were performed via the Bonferroni-adjusted Mann–Whitney U test. A *p* < 0.05 was accepted as significant.

## 3. Results

### 3.1. Geographical Distribution of Participants

Although 4540 people were interviewed, the study was completed with 4539 people because the data of one participant was entered incorrectly. The study was conducted across Turkey according to NUTS-II. Hence, 27.7% of the participants were from the Istanbul Region, 14.0% from the West Anatolian Region, 12.8% from the Aegean Region, 11.1% from the Mediterranean Region, 9.0% from the East Marmara, 7.2 from South-eastern Anatolia Region, 4.4% from West Black Sea, 4.3% from West Marmara, 3.1% from Central Anatolia, 3.0% from Middle East Anatolia, 2.0% from Northeast Anatolia, and 1.5% were from the Eastern Black Sea region (Table 1).

### 3.2. Sociodemographic Characteristics

The distribution the sociodemographic characteristics of the participants is shown in Table 2. Accordingly, the median age of the participants is 40 (18–73), 50.7% are male, 38.6% are associate degree/undergraduate degrees. Furthermore, 68.2% of the participants stated that they did not have a chronic disease, 79.0% did not smoke, and 77.5% stated that they had at least one child.

### 3.3. Perception of Participants Regarding COVID-19 and Vaccines

The distribution of the answers given by the participants to the COVID-19, COVID-19 vaccine, and general immunization questions are shown in Table 3. In total, 20.4% (*n* = 927) of the participants stated that they were exposed to COVID-19 and 97.1% (*n* = 900) of them stated that they used one or more of the home treatment methods. In general, 52.6% (*n* = 2235) of 4239 (93.4%) participants who had been vaccinated for COVID-19 stated that they had received three or more doses of the vaccines. However, 31.0% (*n* = 1313) of the participants stated that they contracted COVID-19 following COVID-19 vaccine. Of the 1313 participants exposed to COVID-19 after vaccination, 76.5% (*n* = 1005) stated that they had BioNTech™ (Pfizer) 19.3% (*n* = 253) Sinovac™ (Coronavac), and 4.2% (*n* = 55) both vaccines. A total of 48% of the participants stated that they had COVID-19 after the first dose of vaccine, 29.5% after the second dose of vaccine, 22.4% after the third dose of vaccine, and 0.2% after the fourth dose of vaccine.

Although 61.7% (*n* = 2799) of the participants stated that they thought the COVID-19 vaccines were protective, 58.4% (*n* = 2651) stated that they were hesitant about the COVID-19 vaccines. Although 98.0% (*n* = 3445) of the participants had their children vaccinated, 19.6% (*n* = 888) stated that they had hesitations about the vaccination of the children. In our study, 47.1% (*n* = 2136) of the participants answered positively to the question of whether vaccination programs should be made compulsory by the government.

### 3.4. Analysis of Variables Affecting VHS-P Scale Scores

The comparison of VHS-P scale scores according to the sociodemographic characteristics of the participants is shown in Table 4. The median score of the VHS-P scale was significantly lower for those over 51 years of age (median scores: 27 vs. 28 vs. 28; *p <* 0.001), and the median score for female participants (median scores: 28 vs. 27; *p <* 0.005). On the other hand, those without children had significantly higher VHS-P scale scores (median scores: 27 vs. 29; *p <* 0.001). The median score of VHS-P in those with chronic diseases was considerably lower (median scores: 27 vs. 28; *p <* 0.001).

The comparison of the VHS-P according to variables (COVID-19 and vaccination) of the participants is shown in Table 5. The median scores on the VHS-P were significantly higher for those who did not have the COVID-19 vaccine (median scores: 27 vs. 43; *p <* 0.001), who did not think that the COVID-19 vaccine was protective (median scores: 25 vs. 33; *p <* 0.001), and who had hesitations about the COVID-19 vaccine (median scores: 31 vs. 23; *p <* 0.001). Those who did not have a vaccination for their children (median scores: 27 vs. 32; *p <* 0.001) and who hesitate about vaccination of children (median scores: 30 vs. 27; *p* < 0.001) had a significantly higher median score on the VHS-P scale.

### 3.5. Analysis of Variables Affecting General AVS-LF Scale Scores 

The comparison of the AVS-LF scale scores according to the sociodemographic characteristics of the participants is shown in Table 4. The median score of the AVS-LF scale of the participants who were under 30 years of age (median scores: 55 vs. 53 vs. 52; *p <* 0.002) and had no children was significantly higher (median scores: 53 vs. 56; *p <* 0.001). There was no significant difference between the median scores according to gender and chronic disease status of the participants. 

The comparisons of the AVS-LF scale scores according to various variables (COVID-19 and vaccination) are shown in Table 5. Those who did not receive the COVID-19 vaccine (median scores: 53 vs. 62; *p <* 0.001), did not think that the COVID-19 vaccine was protective (median scores: 47 vs. 62; *p <* 0.001), and were hesitant about the COVID-19 vaccine (median scores: 59 vs. 44; *p <* 0.001), had a significantly higher median score on the AVS-LF scale. Those who did not have their children vaccinated (median scores: 53 vs. 61; *p <* 0.001) and who were hesitant about childhood vaccinations (median scores: 62 vs. 51; *p <* 0.001), had a significantly higher median score on the AVS-LF scale.

### 3.6. Total and Sub-Dimensional Reliability Analysis of VHS-P and AVS-LF Scales 

The median AVS-LF scale score of the participants was calculated as 54 (95% CI = 54–55). The median scores of the AVS-LF scale subdimension on “benefits and protective value of the vaccine”, “anti-vaccination”, “solutions for non-vaccination”, and “legitimization of vaccine hesitancy” sub-dimensions were calculated as 11 (95% CI = 11–12), 20 (95% CI = 20–21), 12 (95% CI = 12–13), and 10 (95% CI = 10–11), respectively. Cronbach’s alpha reliability and internal consistency coefficient for 21 items of the AVS-LF scale was calculated as 0.916. When the four sub-dimensions of the AVS-LF scale were evaluated separately, Cronbach’s alpha reliability, and internal consistency coefficient values obtained were ranked as benefits and protective value of the vaccine (α = 0.948), anti-vaccination (α = 0.910), legitimization of vaccine hesitancy (α = 0.791), and solutions for non-vaccination (α = 0.767).

The median VHS-P scale score of the participants was calculated as 28 (95% CI = 28–29). The median scores for the “lack of confidence” and “risk” sub-dimensions of the VHS-P scale were calculated as 21 (95% CI = 21–22) and 6 (95% CI = 6–7), respectively. Cronbach’s alpha reliability and internal consistency coefficient for 10 items of the VHS-P scale was calculated as 0.815. When the two sub-dimensions of the VHS-P scale were evaluated separately, Cronbach’s alpha reliability and internal consistency coefficients were ranked as risk (α = 0.856) and lack of confidence (α = 0.838). The Cronbach’s alpha reliability and internal consistency coefficients for 33 items of the two scales were calculated as 0.934 which shows that the participants perceived both scales correctly. 

## 4. Discussion

Vaccine hesitancy was shown to be a reality that existed in a relatively small part of society but did not affect the general population. However, during the pandemic period, vaccine hesitation has become an important issue in the world agenda because the development and production processes of vaccines and rapid approval for use have been exaggerated in the mass media. Ensuring confidence in vaccines is important for individuals to accept the process. It is necessary to evaluate the perception of individuals to understand vaccine acceptance in society and to ensure the adoption of new vaccines such as COVID-19 vaccines. In this study, the perspective of the Turkish population on the COVID-19 vaccine and general immunization programs was evaluated.

In our study, 93.6% of the participants had the COVID-19 vaccine, and 58.4% stated that they had hesitations about COVID-19 vaccines. In a study (*n* = 32,361) conducted in the United Kingdom, 16% of respondents stated that they had a high level of distrust of the COVID-19 vaccine [20], whereas in a study (*n* = 2525) conducted by Salmon et al. in USA, [21] showed that 45% of the participants stated that they had concerns about the content of COVID-19 vaccines. Studies conducted in Turkey have shown that the level of hesitation against the COVID-19 vaccine is variable ranging between 9.7% to 20.9% [22,23,24]. Research shows that intentions for COVID-19 vaccination vary significantly between countries [25]. Although the rate of vaccination for COVID-19 was high in our study, we can say that the level of hesitation in vaccination is higher compared to other studies. Among many reasons for this may be related to the fact that these studies were conducted at different countries and at different period of times from the beginning of the pandemic, the difference in number of cases in different countries, the protective instinct of individuals for their parents and children. Our results also showed that showed that if individuals who have hesitations about vaccination are informed in a correct and comprehensive way, their hesitations can be eliminated.

In our study, 19.6% of the participants stated that they had hesitations about childhood vaccinations. In a study conducted with 1087 parents in Turkey [26], it was shown that 9.3% of them had hesitations about vaccinations. In another study conducted with 477 people in Albania [27], 17% had hesitations about vaccinations. In a study conducted with 3130 people in Italy [28], 15.6% of families had hesitations about vaccinations. In a high-volume study (*n* = 5736) conducted in 18 European countries [29], it was reported that 24% of parents were hesitant about the vaccination of children, while another study with 65,819 participants in 67 countries [30] showed that 13.0% of families did not find vaccines safe. The rate of hesitation about vaccines of the participants in our study was similar to the previous studies.

In our study, the median total score of the AVS-LF scale was found to be 54. It has been reported that there is no cut-off value in the original and Turkish versions of the scale whose validity and reliability have been analyzed, and that as the scale score increases, vaccine hesitancy increases [19]. Accordingly, when the total score that can be obtained from the scale is calculated as 105, we can say that the participants in our study had a moderate anti-vaccination attitude. The median of the VHS-P scale total score was calculated as 28. High scores on this scale also indicate that vaccine hesitancy is high and thus, we can say that the participants have moderate vaccine hesitancy.

Table 4 and Table 5 show whether the scores obtained from the VHS-P and AVS-LF scales used in this study vary according to the sociodemographic characteristics and COVID-19-related characteristics of the participants. We have found that the median scores obtained from the VHS-P and AVS-LF scales of the participants over the age of 51 were significantly lower than the younger individuals. On the other hand, it was observed that the VHS-P and AVS-LF scale score medians were significantly higher in women and in individuals without children. Although there are studies [31,32,33,34] that found a significant difference in vaccine hesitancy according to age and gender, the studies in the literature show that this issue is controversial and highly variable [35,36]. In our study, we have shown that there may be many reasons that affect vaccine hesitancy. All these emphasize the differences in the characteristics of societies or countries. It is understandable that there is lower hesitation about vaccination in the elderly. It has been shown that elderly people died more frequently during the pandemic which may be an important reason for higher acceptance of vaccination programs because vaccination has been shown to reduce mortality [37]. Participants with chronic diseases and smoking habits have a more positive attitude toward vaccination programs due to similar concerns.

In the present study, the median VHS-P and AVS-LF scale scores of those who did not receive the COVID-19 vaccine, who did not think that the COVID-19 vaccine was protective, and who had hesitations about the COVID-19 vaccine were significantly higher. Our results have been supported by a study conducted in Turkey (*n* = 96) [23], the sub-dimension and total score averages of the AVS-LF scale were found to be significantly higher for people who are afraid of getting vaccinated and do not want to be vaccinated. Whereas, in a study conducted with adolescents in Turkey (*n* = 303) [38] showed that the scores of both scales were significantly higher in those who did not want to be vaccinated.

In our study, it was determined that the median VHS-P and AVS-LF scores of those who did not have their children vaccinated and who were hesitant about vaccinations in general, including childhood vaccinations, were significantly higher. Similarly, in a study conducted in Saudi Arabia (*n* = 270) [39], it was shown that one-quarter of the participants had hesitations about childhood vaccinations. Furthermore, in a national study conducted with 2176 people in the USA [40], 68% of the individuals who were hesitant about vaccination postponed or refused routine vaccination of their children. It is a natural result that people who are worried about vaccination do not want to vaccinate themselves or their children and they usually postpone it. Existing causes of concern need to be identified and addressed. Healthcare professionals should use non-confrontational motivational interviewing techniques to understand this situation and to address people’s concerns for vaccination.

One of the most important results of this study is the high reliability (internal consistency) coefficients, which show that the participants gave informed and consistent answers to the survey questions. The internal consistency coefficient of the agreement between the responses given to all items of the VHS-P and AVS-LF scales was calculated as 0.934, which is higher than the alpha coefficients measured in all versions of the scales. Again, when the items belonging to the VHS-P and AVS-LF scales were examined separately, the internal consistency coefficients were calculated as 0.815 and 0.916, respectively, and these results fall into the excellent and/or good categories. Therefore, this study is also the first to test whether the VHS-P and AVS-LF scales can be used safely in the Turkish population.

This study has a few limitations. The first limitation is that this study was conducted with limited financial support. Therefore, many sociodemographic characteristics could not be included in the study due to increased cost. Secondly, the income variable, which is one of the indirect indicators of the status of public health could not be evaluated in the study. Although this variable was originally present in the questionnaire, this variable was excluded from the study due to a problem in the perception of sharing personal income or household income among the participants. The third limitation is that although 98% of the participants vaccinated their children, 19.6% still have concerns about childhood vaccinations. In this study, we could not go into the depths of this problem, but we think that new studies should be done to explain this issue in detail.

## 5. Conclusions

In order to control epidemics, and avoid vaccine-preventable diseases and deaths, it is necessary to eliminate social hesitations about vaccination and increase confidence in vaccines. For this, necessary measures should be taken in society, training should be organized. Furthermore, sufficient and up-to-date, and explanatory information should be provided to the public. It is necessary to make legal arrangements regarding childhood vaccinations and to take necessary measures to ensure immunization of society during pandemics. The source of the existing concerns should be clearly seen, and remedial interventions should be made. Healthcare managers should keep in mind the factors that contribute to vaccine hesitations when planning and developing strategies for vaccination and public health messages.

## Figures and Tables

**Table 1:** Distribution of participants according to geographical regions of Turkey.

Geographical Regions	*n*	%
Istanbul Region	1257	27.7
Western Anatolia Region	635	14.0
Aegean Region	579	12.8
Mediterranean Region	502	11.1
East Marmara	408	9.0
Southeast Anatolia Region	326	7.2
West Blacksea	198	4.4
West Marmara	196	4.3
Middle Anatolia	142	3.1
Middle East Anatolia	138	3.0
Northeast Anatolia	91	2.0
Eastern Black Sea Region	67	1.5

**Table 2:** The sociodemographic characteristics of the participants.

Sociodemographic Characteristics	*n*	%
Age groups		
≤30 years	1435	31.6
31–50 years	1943	42.8
≥51 years	1161	25.6
Gender		
Male	2303	50.7
Female	2236	49.3
Educational level		
Illiterate	88	1.9
Elementary school	461	10.2
Middle School	557	12.3
High school	1562	34.4
Associate/Undergraduate	1752	38.6
Master/PhD	119	2.6
Children		
Yes	3516	77.5
No	1023	22.5
Concomitant chronic diseases		
Yes	1445	31.8
No	3094	68.2
Smoking habit		
Yes	953	21.0
No	3586	79.0

**Table 3:** Distribution of answers of the participants to questions about COVID-19 infection, COVID-19, and routine vaccination of children.

Questions about COVID-19 and Vaccine Awareness	*n*	%
Exposure to COVID-19		
Yes	927	20.4
No	3612	79.6
Management of COVID-19		
In house	900	97.1
Stay in hospital (service)	14	1.5
Stay in hospital (ICU)	8	0.9
Stay in hospital (ICU- intubated)	5	0.5
Vaccination for COVID-19		
Yes	4239	93.4
No	300	6.6
Doses of COVID-19 vaccines		
1 dose	210	5.0
2 doses	1794	42.4
3 doses	2153	50.8
4 doses	82	1.8
Postvaccination exposure to COVID-19		
Yes	1313	31.0
No	2926	69.0
Belief in the protective capacity of the vaccines		
Yes	2799	61.7
No	1740	38.3
Hesitancy towards COVID-19 vaccines		
Yes	2651	58.4
No	1888	41.6
Vaccination status of the children		
Yes	3445	98.0
No	71	2.0
Hesitation towards the vaccination of the children		
Yes	888	19.6
No	3651	80.4
Approval of the legal obligation of vaccination		
Yes	2136	47.1
No	1405	31
No idea	998	22

**Table 4:** Comparisons of VHS-P and AVS-LF scales according to the sociodemographic characteristics of the participants.

Variables (Median (95% CI))	VHS-P	*p*	AVS-LF	*p*
Age (yr)		<0.001 *		0.002 *
≤30 years	28 (28–29) ^a^		55 (55–57) ^e^	
31–50 years	28 (28–29) ^a^		53 (53–54) ^f^	
≥51 years	27 (27–28) ^b^		52 (51–54) ^f^	
Gender		<0.001 ^#^		0.100 ^#^
Male	27 (27–28)		53 (53–54)	
Female	28 (28–29)		54 (54–55)	
Educational level		0.005 *		<0.001 *
Illiterate	26 (24–28) ^c^		49 (46–53)	
Elementary school	27 (27–29) ^c^		53 (52–56)	
Middle School	27 (27–29)		54 (53–56)	
High school	28 (28–29) ^d^		55 (55–57) ^g^	
Associate/Undergraduate	27 (27–28) ^c^		53 (53–54) ^h^	
Master’s/PhD	27 (26–29)		55 (54–58)	
Children		<0.001 ^#^		<0.001 ^#^
Yes	27 (27–28)		53 (53–54)	
No	29 (29–30)		56 (55–58)	
Concomitant chronic disease		<0.001 ^#^		0.102 ^#^
Yes	27 (27–28)		53 (53–54)	
No	28 (28–29)		54 (54–55)	
Smoking habit		0.412 ^#^		0.509 ^#^
Yes	27 (27–29)		55 (54–57)	
No	28 (28–29)		53 (53–54)	

The data are summarized as median (95% confidence interval). a is significantly different from b; c is significantly different from d; e is significantly different from f, and g is significantly different from h; a, b, c, d, e, g, h: the letters represent statistical significance relative to each other in the relevant column (Bonferroni adjusted Mann–Whitney U test; *p* < 0.05); * Kruskal–Wallis H test; # Mann–Whitney U test; AVS-LF: Anti-vaccination scale—long form; VHS-P: Vaccine hesitancy scale adapted to the pandemic.

**Table 5:** Comparison of VHS-P and AVS-LF scales according to COVID-19 and vaccination perspective.

Variables (Median (95% CI))	VHS-P	*p*	AVS-LF	*p*
Exposure to COVID-19		0.339 ^#^		0.682 ^#^
Yes	27 (27–28)		54 (53–55)	
No	28 (28–29)		54 (54–55)	
Vaccination for COVID-19		<0.001 ^#^		<0.001 ^#^
Yes	27 (27–28)		53 (53–54)	
No	34 (32–36)		62 (60–63)	
Belief in the protective effects of the vaccines		<0.001 ^#^		<0.001 ^#^
Yes	25 (25–26)		47 (47–48)	
No	33 (33–34)		62 (62–63)	
Hesitancy for COVID-19 vaccines		<0.001 ^#^		
Yes	31 (31–32)		59 (59–60)	<0.001 ^#^
No	23 (23–24)		44 (44–45)	
Approval of the legal obligation of the vaccination for COVID-19		<0.001 *		<0.001 *
Yes	24 (24–25) ^a^		46 (46–47) ^d^	
No	33 (33–34) ^b^		63 (63–65)^e^	
No idea	29 (29–30) ^c^		55 (55–57)^f^	
Vaccination of the children		<0.001 ^#^		<0.001 ^#^
Yes	27 (27–28)		53 (53–54)	
No	32 (30–36)		61 (59–75)	
Hesitation for vaccination of the children		<0.001 ^#^		<0.001 ^#^
Yes	30 (30–31)		62 (61–63)	
No	27(27–28)		51 (51–52)	

The data are summarized as median (95% confidence interval). a, b, and c are significantly different from each other; d, e, and f are significantly different from each other; a, b, c, d, e, f: the letters represent statistical significance relative to each other in the relevant column (Bonferroni adjusted Mann–Whitney U test; *p* < 0.05); * Kruskal–Wallis H test; # Mann–Whitney U test; AVS-LF: Anti-vaccination scale—long form; VHS-P: Vaccine hesitancy scale adapted to the pandemic.

## Data Availability

The datasets analyzed during the current study are available from the corresponding author upon reasonable request.
